# Missed Testing Opportunities for HIV Screening and Early Diagnosis in an Urban Tertiary Care Center

**DOI:** 10.1155/2017/5708620

**Published:** 2017-07-04

**Authors:** Joseph DeRose, Jason Zucker, David Cennimo, Shobha Swaminathan

**Affiliations:** ^1^Department of Medicine, Rutgers New Jersey Medical School, Newark, NJ, USA; ^2^Departments of Medicine and Pediatrics, Division of Infectious Diseases, Columbia University Medical Center, New York, NY, USA; ^3^Departments of Medicine and Pediatrics, Division of Infectious Diseases, Rutgers New Jersey Medical School, Newark, NJ, USA; ^4^Department of Medicine, Division of Infectious Diseases, Rutgers New Jersey Medical School, Newark, NJ, USA

## Abstract

Newark, New Jersey, is disproportionally affected by HIV with one of the highest prevalence rates in the United States. Rutgers New Jersey Medical School is a major healthcare provider to Newark's underserved population and has implemented a HIV testing program that can diagnose and link newly diagnosed individuals to care. We conducted a retrospective chart review of all new patients seen in the Infectious Disease Practice from January 1, 2013, to December 31, 2014, to determine the proportion of patients with a missed testing opportunity (MTO) (patients with a new HIV diagnosis with an encounter at the institution in the 1 year prior to their first appointment). 117 newly diagnosed patients were identified. 36 (31%) had at least one MTO. A total of 34 (29%) of newly diagnosed patients had AIDS at presentation and 17% had CD4 counts of 50 cells/*μ*L (*p* value 0.5). The two most common locations of a missed testing opportunity were the hospital ED (45%) and subspecialty clinics (37%). This study demonstrates that, even in a high prevalence institution with HIV counseling, testing, and referral service, HIV screening is lacking at multiple points of care and patients are missing opportunities for earlier diagnosis and treatment.

## 1. Introduction

The Centers for Disease Control and Prevention (CDC) estimates that over 150,000 people living in the United States with human immunodeficiency virus (HIV) are unaware of their diagnosis [1]. In addition, there is growing evidence that initiating antiretroviral therapy (ART) early can reduce HIV viral load as well as the number of infections and can help reduce HIV transmission at the population level [2]. Access to free HIV testing services is available in all fifty of the United States and Washington DC [3]. Despite the improvement and availability of HIV testing centers and programs to facilitate linkage to care and treatment, 49% of those testing positive for HIV are not in care and an additional 11% of those in care are not receiving ART [4]. Of the patients receiving ART, only 30% are able to achieve adequate HIV virologic suppression [4]. Many of these challenges can contribute to the approximately fifty thousand new HIV infections per year in the United States [1].

All routine clinical encounters represent an opportunity for HIV screening and thereby earlier identification and prior studies have shown that missed testing opportunities occur in multiple healthcare settings including emergency departments, medical subspecialty clinics, and outpatient pharmacies [5, 6]. Reducing the number of these missed testing opportunities for identifying people living with HIV, linking them to care, and initiating ART represent a major step in ending the HIV epidemic. The United States Preventive Services Task Force and the Centers for Disease Control both recommend one time screening of adolescents and adults aged 15–65 years for HIV infection with repeated screening indicated in patients at high risk and in high prevalence settings [7, 8].

The state of New Jersey ranks 4th in terms of the number of people living with HIV/AIDS with more than 37,000 statewide [9, 10]. University Hospital (UH) and Rutgers New Jersey Medical School (NJMS) are located in Newark, New Jersey, an area which resides in the center of one of the highest prevalence HIV cities in the United States. Newark has an HIV prevalence 8 times that of New Jersey and among the highest rates (2%) in the United States [11, 12]. The prevalence rate in Newark is ever higher than 3% among non-Hispanic Black and African Americans [10]. As part of their mission, University Hospital and Rutgers NJMS have access to a state funded on-site rapid HIV counseling, testing, and referral service to expedite testing and linkage to care of high risk individuals with over 80% of patients linked to care following positive results. In an effort to identify missed testing opportunities for HIV screening, we evaluated whether newly diagnosed patients with HIV had opportunities for HIV testing and where those missed testing opportunities occurred.

## 2. Methods

We conducted a retrospective chart review study in which we reviewed patient records from the Rutgers New Jersey Medical School Infectious Disease Practice (IDP) located at University Hospital, Newark. The IDP is the single largest provider of HIV primary care in Newark, NJ, and the majority of patients tested within UH are referred to the IDP for care and treatment. All new patients seen in the IDP from January 1, 2013, to December 31, 2014, were included in the analysis. “New patients” were defined as patients 18 years of age and older with no previous documentation of HIV infection or designated as “newly diagnosed” in the initial clinic visit documentation. Review of medical records was conducted using outpatient Centricity Electronic Medical Record (EMR) and inpatient Epic EMR.

Data collected included age, gender, self-identified race, date of visit, previous known history of HIV infection, location of positive HIV test, risk factors for acquiring HIV, date of diagnosis, CD4 count at diagnosis, viral load at diagnosis, type of positive test, and most recent prior negative test. The patient's chart was then reviewed to see if they had a visit in University Hospital or a Rutgers NJMS affiliated clinic and whether or not HIV testing was offered or performed in the twelve months prior to their IDP clinic visit.

A “missed testing opportunity” was defined as any visit to University Hospital or a Rutgers NJMS affiliated clinic in the twelve-month period prior to the first outpatient IDP visit in which they were not tested for HIV infection. Only visits to Rutgers NJMS and affiliated clinics were included in the analysis.

Data entry was conducted using Microsoft Excel 2010 (Microsoft Corporation, Redmond, WA). Univariate analyses were conducted to look at factors associated with missed opportunities for HIV testing. This project was approved by the Rutgers Biomedical and Health Sciences Institutional Review Board.

## 3. Results

We reviewed a total of 314 patients and identified 117 newly diagnosed patients.  Table 1 demonstrates the demographics of newly diagnosed patients. The mean age of the patients was 37 years (range 18–68). The most common self-identified race was African American (71%), followed by Hispanic (6%). The majority of patients were men (62%) and the most common risk factor recorded was heterosexual sex (50%).

Of the 117 newly diagnosed patients, 36 (31%) had at least one missed testing opportunity for screening and earlier diagnosis. The total number of missed testing opportunities was 126 with a mean of 3.6 missed testing opportunities per patient (range 1–20). The median HIV RNA of the newly diagnosed patients was 225,910 copies/mL, the median CD4 was 357 cells/*μ*L, and 29% had AIDS at presentation. The majority of patients (77%) were tested in the outpatient setting.

Table 2 compares the characteristics among patients with a missed testing opportunity versus those without. It shows that although there were no statistically significant differences between the two groups, there was a clinically meaningful difference within the group with missed testing opportunities as they had a lower CD4 count. The mean viral load at diagnosis was 38% higher for patients with a missed testing opportunity than for those without one (*p* value 0.36). A total of 34 (29%) of newly diagnosed patients had AIDS at presentation and 17% had CD4 counts of 50 cells/*μ*L (*p* value 0.5). A total of 23 (28%) patients with no missed testing opportunity had an initial CD4 count of <200 cells/*μ*L while 11 (31%) patients with a missed testing opportunity had an initial CD4 count of <200 cells/*μ*L (*p* value 0.36).  Table 3 demonstrates the difference in the numbers of missed testing opportunities by location. The two most common locations of a missed testing opportunity were the hospital emergency department (45%) and subspecialty medical clinics (37%).

## 4. Discussion

Earlier diagnosis and treatment of HIV have led to substantial benefits including reduction in rates of transmission, adoption of safer behaviors, and a decrease in both morbidity and mortality [13–16]. Implementation of policies advocating for increased HIV testing, linkage to care, and early ART are key factors responsible for the decreases. The START study recently showed that HIV infected individuals have a considerably lower risk of developing AIDS or other serious complications when antiretrovirals are started earlier in their disease course, even when they are asymptomatic and have higher CD4 counts [16]. Furthermore, previous studies have demonstrated that earlier initiation of therapy not only limits complications but also reduces the rates of HIV transmission [16, 17]. For those reasons, it is critical that all PLWH (persons living with HIV infection) be diagnosed, linked to care, and started on antiretroviral therapy as early as possible. At our institution, despite the availability of on-site HIV testing, there were many patients who had >1 missed testing opportunity. This could certainly account in part for the large portion of patients (29%) who were seen with AIDS at the time of their new HIV diagnosis. Another explanation for the number of patients seen with AIDS at the time of diagnosis may be a population of “late testers.” Many reasons for “late testing” have been hypothesized in the literature [18] including late testing being more common in groups who do not consider themselves at high risk for infection (e.g., heterosexual contact acquisition). In our patient population, the most common risk factor for a patient with a missed testing opportunity was heterosexual contact further strengthening the concern for early and appropriate testing for patients. Routine screening for HIV in all health care encounters is one way to lead to better outcomes allowing patients who are unaware of their disease to be diagnosed and linked to care earlier on in their illness.

New HIV infections are driven by failures in all aspects of the HIV treatment cascade. This study demonstrates that, despite increased local prevalence and the widespread availability of on-site HIV testing, many patients continued to have missed testing opportunities for screening and linkage to care.

The most common site of missed testing opportunities in our study was the emergency department. A recent study revealed that approximately 80% of adults who had visited the emergency department did so because of a lack of access to other providers [19]. University Hospital plays a vital role in providing care to the underserved population. As the University Hospital is the largest provider of charity health care in New Jersey, it remains critical to acknowledge that these patients were missing opportunities for screening at what may have been their only exposure to the healthcare system. While reasons for lack of screening in our emergency department are unclear, results can likely be extrapolated to fit multiple prior studies which attributed lack of time, lack of resources, and concerns regarding privacy and follow-up care to poor screening compliance [20, 21]. In an effort to help address some of these issues, Gaydos et al. hypothesized that enrolling patients in a self-testing program may help to overcome some of the limited time and resource constraints that arise in busy emergency departments [22]. About half of the patients approached consented to enrollment in the program and over 98% completed the test. While only one new diagnosis of HIV was found with the testing, the program provides guidance for emergency departments in ways to overcome the constraints that are currently in place given the current structure testing. It should be noted that Rutgers and University Hospital have a free voluntary HIV screening program where patients can be referred 5 days a week for testing. This is in addition to the dedicated personnel in the emergency department that are available 14 hours per day, 7 days per week, to perform testing. This program is designed to increase the number of patients tested and to link them to care once a diagnosis of HIV is confirmed. We have previously shown that the majority of patients tested in the ED were in the “Fast Track” area of the ED where patients are generally more clinically stable and less likely to have life threatening conditions. Given the results of this and other studies looking at HIV screening rates in our emergency department, we have now implemented a routine “opt-out” HIV testing program in the ED. This strategy involves an automatic HIV test being ordered at triage provided the patient does not have a history of one of the following: current HIV infection or recent (within 12 months) HIV test or the patient declines testing. We will be assessing the impact of the routine HIV testing program in coming studies.

The second most common site of missed testing opportunities in our study was subspecialty medical clinics. As mentioned above, Rutgers and University Hospital have a dedicated program designed to identify and test patients at risk for HIV. This program is not only available in our emergency department but also available in our medical subspecialty and dental clinics during weekday hours. It is the sole responsibility of these providers to test patients and link them to care and while concrete data is not available from the program, these providers report the overwhelming majority of patients that accept testing when offered. It is also standard practice in the program that patients with positive results are referred to the clinic that same day or the next business day if needed. Our study showed that there were a large proportion of patients with MTO in medical subspecialty clinics. Possible explanations include lack of the subspecialty provider's knowledge of the available program, lack of knowledge of the CDC screening guidelines, or the fact that providers may be uncomfortable discussing HIV status with their patients. It is also possible that specialists may not consider HIV testing to be their responsibility. In an area such as Newark, where patients may not have access to primary care routinely, every effort should be made to offer HIV testing including at the subspecialty clinics. Studies have supported these explanations showing that the number one reason for lack of screening was a lack of knowledge of the guidelines followed by a concern regarding arranging follow-up for patients who tested positive [23]. Given the resources available at our institution, educating providers of the dedicated program may lead to significant improvements in screening rates. We have used this data to improve education of specialty clinicians and highlight the same day linkage to care policy to ensure that thereby addressing any concerns they may have.

Although many of the patients analyzed did not have a missed testing opportunity, those that did had multiple chances to be evaluated with a mean number of missed testing opportunities per patient of 3.6. Patients with a missed testing opportunity were also more likely to present with a lower CD4 at presentation than those without (*p* value 0.36). While there was no statistical difference in overall CD4 counts among patients with a missed testing opportunity compared to those without, it remains clinically relevant as patients with lower CD4 are more susceptible to a number of complications. It is also possible that our study population may not have been large enough to detect a difference or that the time frame of one year may not be adequate enough to elicit a statistical difference.

Limitations of the study include the fact that there are 4 major hospitals, each with their own emergency department and inpatient facilities within a 4-mile radius of our institution, thereby affecting the number of people presenting to our facilities which may result in an underestimation or overestimation of missed testing opportunities. Additionally, no information was available regarding outside testing done by other institutions such as the New Jersey Health Department or Planned Parenthood. While we recognize this as a limitation, all of the patients that were tested and referred to the IDP denied knowledge of preexisting HIV infection so, despite the possibility of prior testing, they were unaware of their diagnosis when questioned. It also would have been unlikely that they would have been tested previously and had been unaware of a positive result given that these institutions use point of care testing funded by the state Department of Health and therefore would have been informed of their test results at that time.

Despite the fact that our institution has on-site HIV testing and established programs to help connect HIV positive patients to care, many patients still had multiple missed testing opportunities for screening. If these patients were afforded the opportunity of entering the HIV continuum of care earlier in their disease course, their outcomes could be improved. Patients would also be less likely to continue to spread the disease given their reduced viral loads while on treatment. Education and close working relationships between the infectious disease department and the emergency department is crucial to encourage routine testing and prevent further missed opportunities for early HIV diagnoses. Finally, this paper provided the basis to propose a change within the institution in regard to its HIV testing model. The institution now operates an HIV testing model with a testing-in policy that is more closely aligned with CDC recommendations for opt-out screening. Further studies are required to elicit if this can help with earlier identification of HIV positive patients.

## Figures and Tables

**Table 1:** Baseline characteristics of newly diagnosed HIV patients at University Hospital HIV Clinic, Newark, New Jersey, 2013-2014.

	*N* = 117 (37%)
*Mean age*	37
*Gender*	
Male	72 (62%)
*Self-identified race*	
African American	83 (71%)
Hispanic	7 (6%)
White	5 (4%)
Others	18 (15%)
Unknown	4 (3%)
*HIV risk factor*	
Heterosexual	59 (50%)
MSM	36 (31%)
Injection drug use	8 (7%)
Bisexual	4 (3%)
Transfusion	3 (3%)
Unknown	4 (3%)
Commercial sex worker	1 (1%)
Other	2 (2%)
*Site of diagnosis*	
Inpatient	27 (23%)
Outpatient	90 (77%)
*HIV RNA > 100,000 copies/mL *	34 (29%)
*CD4 < 200 cells/μL*	34 (29%)
*CD4 < 50 cells/μL*	20 (17%)

MSM: men who have sex with men.

**Table 2:** Comparison of newly diagnosed patients with and without missed testing opportunities at University Hospital HIV Clinic, Newark, New Jersey, 2013-2014.

	Missed testing opportunities, *N* = 35 (%)	No missed testing opportunities, *N* = 82 (%)	*p* values
*Age*	39	36	
*Gender*			
Male	22 (63)	50 (61)	
Female	13 (37)	32 (39)	
*Self-identified race*			
African American	27 (77)	56 (68)	
Hispanic	2 (6)	5 (6)	
Caucasian	1 (3)	4 (5)	
Others	4 (11)	14 **(**17)	
Unknown	1 (3)	3 (4)	
*HIV risk factor *			
Heterosexual	17 (49)	42 (51)	
MSM	10 (29)	26 (32)	
Transfusion	2 (6)	1 (1)	
Intravenous drug use	3 (9)	5 (6)	
Bisexual	1 (3)	3 (4)	
Commercial sex worker	0 (0)	1 (1)	
Other	1 (3)^*∗*^	0 (0)	
Unknown	1 (3)	4 (5)	
*HIV RNA > 100 K copies/mL @ diagnosis*	11 (32)	23 (28)	0.36
*CD4 < 200 cells/µL*	11 (32)	23 (28)	0.36
*CD4 < 50 cells/µL*	6 (17)	14 (17)	0.5
*Site of diagnosis*			
University Hospital ED	5 (14)	16 (20)	0.25
ID clinic	9 (26)	22 (27)	0.45
Primary care clinic	4 (11)	1 (1)	
Subspecialty medical clinic	4 (11)	8 (10)	
External	3 (9)	7 (9)	
Inpatient	4 (11)	19 (23)	
Intensive care unit	2 (6)	2 (2)	
Others	4 (11)	7 (9)	

MSM: men who have sex with men; ED: emergency department; ID: infectious disease; *∗* = occupational exposure.

**Table 3:** Location of missed testing opportunities by location at University Hospital HIV Clinic, Newark, New Jersey, 2013-2014.

Missed opportunities by location		
University Hospital ED	57	45%
ACC, ID clinic	0	0
ACC, subspecialty clinic	46	37%
ACC, primary care clinic	15	12%
University Hospital ICU	1	1%
University Hospital Inpatient	7	6%

ED: emergency department; ACC: ambulatory care center; ID: infectious disease; ICU: Intensive Care Unit.
